# Quantitative structure activity relationship (QSAR) modeling for adsorption of organic compounds by activated carbon based on Freundlich adsorption isotherm

**DOI:** 10.1371/journal.pone.0338483

**Published:** 2025-12-15

**Authors:** Siyi Ding, Yefan Yang, Ting Tan, Qunshan Wei, Zhemin Shen, Qiong Liu, Xinshan Song, Yuhui Wang, Charles Nzila, Christopher W.K. Chow

**Affiliations:** 1 Institute of Artificial Intelligence, Donghua University, Shanghai, China; 2 College of Mechanical Engineering, Donghua University, Shanghai, China; 3 College of Environmental Science and Engineering, Donghua University, Shanghai, China; 4 State Environmental Protection Engineering Center for Pollution Treatment and Control in Textile Industry, Donghua University, Shanghai, China; 5 School of Environmental Science and Engineering, Shanghai Jiao Tong University, Shanghai, China; 6 School of Engineering, Moi University, Eldoret, Kenya; 7 Sustainable Infrastructure and Resource Management (SIRM), UniSA STEM, University of South Australia, Mawson Lakes, South Australia, Australia; Faculty of Science, Alexandria University, EGYPT

## Abstract

The Freundlich isotherm parameters K and 1/n are typically regarded as empirical constants. However, the underlying theoretical basis for the widespread applicability of the Freundlich isotherm in describing adsorption processes for diverse organic compounds remains unclear. In this study, we successfully elucidated the reason by developing two optimal quantitative structure-activity relationship (QSAR) models: one correlating K with quantum chemical parameters and another linking 1/n to these parameters. The modeling results demonstrated that both K and 1/n exhibit strong correlations with specific quantum chemical descriptors, indicating that the empirical Freundlich isotherm’s applicability is fundamentally linked to the molecular structural characteristics of organic compounds. Key quantum parameters influencing K were identified as ∑q(O + N), q(CH+)_max_, ELUMO, Fukui(-)_max_, and Wiberg(C-C)_min_, suggesting that charge distribution, carbon bond energy, and active site energy are the primary factors governing adsorption efficiency on activated carbon. The QSAR model for 1/n yielded similarly novel and consistent insights, showing that the value of 1/n also correlated with molecular structural characteristics. Both models were rigorously validated and confirmed to be stable, robust, and accurate through standard statistical evaluations. These QSAR models can now be employed to identify whether an organic compound would conform to the Freundlich Isotherm and predict the adsorption efficiency of this compound by activated carbon based on their quantum chemical parameters. As to the practical implications, this study provides a convenient reference method for assessing the applicability of activated carbon adsorption in treating emerging organic pollutants in drinking water plants and a theoretical foundation for developing intelligent management systems in water treatment facilities.

## 1 Introduction

In recent years, the widespread use of organic compounds (e.g., halogenated hydrocarbons, benzene, and pollutants containing nitrogen or oxygen functional groups) in industrial and agricultural production has led to their entry into the water environment through the pathways of agricultural runoff and industrial wastewater, which has brought about great risks and impacts on the environment and human health [1,2]. Therefore, it is necessary to effectively control the impact of these organic compounds on drinking water. Activated carbon adsorption (ACA) technology remains employed in drinking water treatment plants worldwide due to its high efficiency, easy operation and stable performance in removing some organic pollutants. However, for some emerging organic compounds, the removal efficiencies by ACA are still not clear, which needs experimental assessment individually. Nevertheless, the experimental assessment of the removal efficiencies for specific pollutants constitutes a labor-intensive process, and most of small or even middle-size drinking water plants don’t have the sufficient analytic instruments and technicians. Therefore, establishing a quantitative structure-activity relationship (QSAR) model based on quantum chemical parameters of organic molecular structures holds significant reference value for current drinking water treatment plants to evaluate their activated carbon units in controlling emerging organic contaminants.

As a well-established cheminformatics methodology, QSAR quantitatively relates measurable biological/chemical activities of compounds to their structural parameters using mathematical models and molecular descriptors [3,4]. Typically, it can reliably predict key compound properties including metabolic activity and toxicity profiles using only molecular descriptors derived from compound structures [5]. Current adsorption models predominantly focus on hydrophobic interactions as the interpretation of adsorption results, particularly for nonpolar or weakly polar organic compounds [6]. Hobbs et al. [7] employed the Freundlich isotherm to develop a QSAR model for aromatic compound adsorption on powdered activated carbon. However, the structural homogeneity of the selected adsorbates limited the universal applicability of the model. Blum et al. [8] utilized molecular connectivity and (X/C)_min_ to develop a QSAR model in order to assess the adsorption of organic compounds (both aromatic and aliphatic) by activated carbon. However, molecular structure quantum parameters were not taken into account, therefore the QSAR model could not fully predict or explain the adsorption properties of these compounds. Rao et al. [9] built three different QSAR models: the octanol-water partition coefficient model (K_ow_ model), the linear solvation energy model (LSE model), and the molecular connectivity indices theory (MCI) model, which were only validated by the experimental adsorption data of Dewulf et al. [10] and Mader et al. [11] and were not correlated with quantum chemical parameters of molecular structures, limiting the model’s applicability. Utilizing partial least squares regression (PLS), Kamlet et al. established multivariate linear regression equations to elucidate specific solute-adsorbent interaction mechanisms. The models integrated three key solvation parameters – dipolarity/polarizability (π*), hydrogen-bond donor acidity (α), and hydrogen-bond acceptor basicity (β) – quantified through systematic analysis of removal kinetics of mono-nitroaromatic by activated carbon adsorption [12]. However, the structures of the compounds studied are relatively homogeneous, and therefore the adsorption mechanism may not be suitable for other kinds of organics with different structures.

According to the summary of existing results shown above, it can be seen that there were few researches establishing QSAR models for the key parameters (K and 1/n) of Freundlich Isotherm, which are commonly considered as empirical constants determining the removal efficiencies of organics by activated carbon adsorption. It is still not very clear why the empirical Isotherm model has been applicable to so many organics for many years.

Therefore, the first aim of this study is to reveal whether the key parameters (K and 1/n) of Freundlich Isotherm are correlated with some of the quantum parameters of organic molecular structure, then to explain why the empirical Isothem model can be applicable for many organic compounds. Is this really a coincidence, or is it a special coincidence that just happens to fall on the track of the laws of the world? If the QSAR model can be constructed successfully, it would be a significant innovation result to let the question get an exact answer. The findings will further reveal which molecular quantum parameters determine the applicability of the Freundlich isotherm,thereby provide novel theoretical insights into this classical adsorption model.

The second aim of this study is to provide a kind of useful calculation model for current drinking water plants to evaluate the possible efficiencies of their activated carbon units when the plants meet various emerging organic compounds. The results would help the plants to overcome their weak points on the analytical instruments and technicians.

In future intelligent water treatment plant management systems, core processes like activated carbon adsorption will require fundamental chemical interaction models and performance evaluation frameworks. This study will develop QSAR models for Freundlich parameters (K and 1/n) of organic compound adsorption on activated carbon, which will significantly contribute to building next-generation smart water management systems.

## 2 Materials and methods

### 2.1 Data sets

Table 1 summarizes the Freundlich adsorption isotherm (Eq. (1)) K and 1/n for these 47 organic compounds, which were extracted from the Contaminant Control List of Drinking Water developed by the U.S. Environmental Protection Agency and related literature [5,8,13–16]. These compounds structurally include halogenated hydrocarbons (especially chlorinated alkanes and aromatic hydrocarbons), benzene rings and their derivatives, and nitrogen or oxygen containing functional groups. All data were obtained from adsorption experiments using Fitrasorb-400 coal activated carbon. The concentrations for all organic compounds during the adsorption experiment were in the same measurable range of the analytical method (normally referred to USA EPA method). Uniform criteria were followed in screening the data: (1) the experimental temperatures were controlled within the range of room temperatures (25°C); (2) the final state data that reached adsorption equilibrium were chosen (contact time was greater than 48 h). The above standardized screening ensured the maximum baseline comparability of the data.

**Table 1:** Fitting parameters of Freundlich adsorption isotherms for 47 organic compounds.

Name	K	1/n	Name	K	1/n	Name	K	1/n
Benzene	1260	0.533	2,4-Dinitrotoluene	96100	0.157	Diquat	2260	0.242
Toluene	5010	0.429	1,3-Dichloropropane	897	0.497	Dinoseb	30400	0.279
o-Chlorotoluene	23200	0.378	1,1-Dichloropropene	2670	0.374	Picloram	23400	0.18
o-Dichlorobenzene	19300	0.378	1,1,1,2-Tetrachloroethane	1070	0.604	Metribuzin	48700	0.193
Ethyl benzene	9270	0.415	1,2-Dibromoethane	888	0.471	Aldicarb	8270	0.402
Styrene	12200	0.479	1,3,5-Trichlorobenzene	63800	0.324	Oxamyl	1740	0.793
p-Xylene	12600	0.418	Dibromochloromethane	585	0.636	Lindane	15000	0.433
p-Chlorotoluene	35900	0.34	trans-1,2-Dichloroethylene	618	0.452	Alachlor	81700	0.257
Bromobenzene	17200	0.364	cis-1,2-Dichloroethylene	202	0.587	Atrazine	38700	0.291
Chlorobenzene	9170	0.348	Bromoform	929	0.665	Carbofuran	16400	0.408
1,2-Dichloroethane	129	0.533	1,1,1-Trichloroethane	335	0.531	Dicamba	33100	0.147
Isophorone	9750	0.271	Bromodichloromethane	241	0.655	Glyphosate	87600	0.119
1,2-Dichloropropane	313	0.597	1,1-Dichloroethene	470	0.515	Metolachlor	98200	0.125
1,2,3-Trichloropropane	1080	0.613	1,1,2-Trichloroethane	365	0.652	Simazine	31300	0.227
tert-Butyl methyl ether	218	0.479	Trichloroethylene	2000	0.482	Cyanazine	102000	0.126
2,4,5-trichlorophenoxy acetic acid	43000	0.21	Dibromochloropropane	6910	0.501	–	–	–

The literature data compiled in this study were obtained from batch equilibrium experiments conducted at concentrations within the measurable range of the analytical methods for most compounds. The pH values of the solutions varied from 5.3 to 8.0 in the data extracted from literature because the adsorption experiments were performed without pH control, in which the organic compounds were dissolved into the pure water directly and kept in their natural states. In addition, the K with excessive variance data was excluded considering the applicability of the data and the stability of the later model. To ensure the robustness of the developed model, we followed this principle in organizing the extracted data from literature that the data conforms to a Normal Distribution (i.e., Gaussian Distribution) were retained but the data deviating from the overall Normal Distribution are excluded.


$$\begin{array}{c} Q_{e}=K{C_{e}}^{\text{1}/n} \end{array}$$ (1)

where Q_e_ is the adsorbed amount at equilibrium, mg/g; C_e_ is the concentration at equilibrium, mg/L; K is Freundlich’s constant; 1/n is the adsorption index. The concentrations for all organic compounds during the adsorption experiment were in the same measurable range of the analytical method (normally referred to USA EPA method).

### 2.2 Calculation method of structural parameters

Studies have shown that chemical structural parameters are closely related to the properties of compounds [17], and thus focuses on common parameters that represent the structural properties of organic compounds, which are shown in S1 Table. These parameters were drawn and calculated using ChemDraw, Gaussian, Material Studio, and Multiwfn [18] software. All calculations were performed using the Density Functional Theory Method (DFT) [4,19]. The DFT calculations of compounds are performed in a consistent identical and theoretical state. The core principle of Density Functional Theory (DFT) calculations lies in the assumption that, under a unified idealized state, the electronic structure of a system (such as electron density, energy levels, and orbital energies) is determined by solving the Schrödinger equation, thereby reflecting the intrinsic properties of the material. This methodology is applicable to diverse systems, including vacuum, solution, and surface environments. The B3LYP method and 6 - 311G (d,p) basis set were chosen to obtain the structural parameters of the substance by Gaussian 09 [20]. The DMol3 module and the GGA-BLYP method were used to calculate the energies of the optimized structures by Material Studio 7.0 [21]. The details of the molecular parameters of each organic compound are shown in S2 Table. It should be noted that all quantum chemical calculations were performed under vacuum conditions. While this approach is standard in QSAR studies for computational efficiency and has been shown to yield robust correlation trends for adsorption affinity, it does not explicitly account for aqueous solvation effects. Future investigations will employ implicit solvation models (e.g., SMD, PCM) and descriptors like solvent-accessible surface area (SASA) to better simulate the aqueous-phase adsorption environment and refine the predictions.

### 2.3 Model validation methods

In this study, QSARINS software was used to build the model using the multiple linear regression method [21,22], and the model was validated by Ordinary Least Squares (OLS) method [23] with Y-randomization and Leave-Many-Out cross-validation method. The quality of the constructed QSAR was evaluated by different evaluation metrics to test its robustness and predictive ability [24]. The information of evaluation indexes is shown in S2 Table.

Y-randomization validation [25,26] is mainly to determine the stability of the model by testing the chance relationship between the dependent and independent variables. This method disrupts the dependent variables firstly and then builds a new model for the randomly disrupted dependent variable and initial independent variable. The process is repeated several times. For a stable QSAR model, R^2^_yrand_ and Q^2^_yrand_ should be lower compared to the original model’s R^2^ and Q^2^.

Leave-Many-Out cross-validation [27,28] was used to study the behavior of the model when many compounds were excluded. Multiple samples are drawn from the data set at a time, and the remaining samples are used to build a model. The drawn samples are predicted by the constructed model. This above process is repeated several times.

### 2.4 Application domains

In this study, a Williams plot [25,29] was applied to visualize the applicable domain. The accuracy of the optimal QSAR model prediction was judged based on the standardized residuals (σ) and leverage ($h_{i}$). The standardized residuals are defined in the following equation (2):


$$\begin{array}{c} \sigma =\frac{\left(y_{i}-y_{i}^{\sim }\right)}{\sqrt{\frac{\sum_{i=\text{1}}^{n}{\left(y_{i}-y_{i}^{\sim }\right)}^{\text{2}}}{n-\text{1}}}} \end{array}$$ (2)

where $y_{i}$ is the experimental value of the compound, $y_{i}^{\sim }$ is the predicted value the compound, *n* is the number of compounds in the training set. The ideal range of σ is (−3, 3). The leverage is defined in the following equation (3):


$$\begin{array}{c} h_{i}=x_{i}{\left(X^{T}X\right)}^{-\text{1}}x_{i}^{T}(i=\text{1},\text{2}\ldots \ldots n) \end{array}$$ (3)

where $x_{i}$ is a vector of descriptor for the compound, X is the descriptor matrix in the training set. The alert leverage value ($h^{*}$) is defined in the following equation (4):


$$\begin{array}{c} h^{*}=\frac{\text{3}(m+\text{1})}{n} \end{array}$$ (4)

where m is the number of predictor variables and *n* is the number of compounds in the training set. It was only ${h_{i}<h}^{*}$ that it was considered compliant.

## 3 Results and analysis

### 3.1 Experimental results of organic compound

In the Freundlich isotherm, the larger the value of K, the higher the adsorption capacity of the adsorbent at a given pressure; and the larger the value of n, the stronger the nonlinearity of the adsorption process. Both determine the shape of the Freundlich isotherm curve and therefore together determine the characteristics of the adsorption process and the performance of the adsorbent. The K and 1/n values of the 47 compounds summarized are shown in Table 1. The K and 1/n values varied from compound to compound. Among the 47 compounds selected, glyphosate had the smallest Freundlich adsorption index 1/n (0.119), corresponding to K of 87,600, and oxamyl had the largest adsorption index 1/n (0.793), corresponding to K of 1740. The difference in the Freundlich adsorption index 1/n of the above two compounds is about 0.674, and the difference in the K value is about 85860. It can be seen that the structural differences of these organic compounds are significant, resulting in a wide range of adsorption properties among them. Therefore, the model constructed on the basis of the above compounds may have wider applicability.

### 3.2 Calculation results of the structure of compounds

The structural parameters of the 47 organic compounds studied are shown in S2 Table. The differences in dipole moments among these 47 organic compounds indicate that the polarities of the organic compounds vary considerably. The total molecular energy (E) represents the interaction of kinetic and potential energy between electrons. Its maximum value is −232.333 (benzene), and the minimum value is −7761.158 (bromoform). There is a 33-fold difference between these two values, which indicates the diversity between the structures of the studied organic compounds.

q(CH+)_max_ has a maximum of 0.198 e (dicamba) and a minimum value of 0.039 e (tert-butyl methyl ether); q(CH+)_min_ has a maximum of 0.129 e (picloram) and a minimum of 0.020 e (metolachlor); q(C-)_max_ has a maximum of 0.219 e (glyphosate) and a minimum of −0.045 e (benzene); q(C-)_min_ has a maximum of 0.034 e (bromoform) and a minimum of −0.137 e (1,2-dichloropropane).∑q(O + N) has a maximum of 0.154 e (diquat) and a minimum of −1.554 e (glyphosate), and 29 compounds did not contain oxygen and nitrogen. ∑q(-)/N_C_ has a maximum of 0.060 e (diquat) and a minimum of −0.061 e (1,2-dichloroethane). These results demonstrate the charge distribution of the organic compounds studied, which is important for analyzing the adsorption of organic compounds by activated carbon.

The Wiberg index [30] is used to quantify the degree of bonding between atoms. This parameter can be used to analyze the bonding properties of molecules and to predict the breaking and formation of chemical bonds [31,32]. Wiberg(C-C)_max_ and Wiberg(C-C)_min_ represent the bonding strengths of the carbon-carbon bond in a molecule. In this study, Wiberg(C-C)_max_ has a maximum of 4.083 (1,1,1,2-tetrachloroethane) and a minimum of 3.893 (1,2-dichloroethane). The maximum of Wiberg(C-C)_min_ is 3.963 (benzene) and the minimum is 1.072 (1,3-dichloropropane).

**Fig 1:**
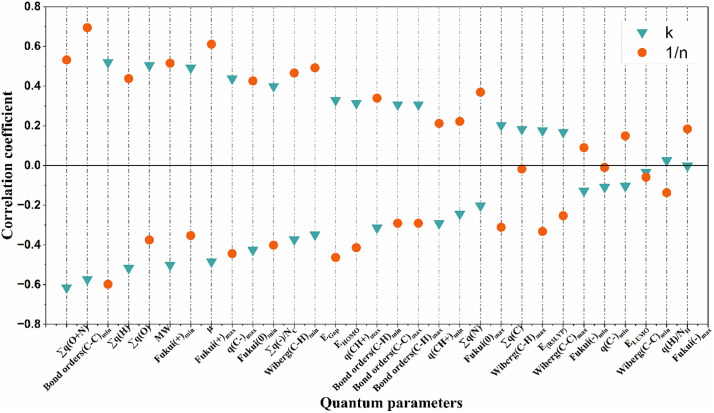
Correlation coefficients of Freundlich parameters (K, 1/n) with 31 molecular parameters.

E_HOMO_ and E_LUMO_ represent the charge transfer that occurs within the molecules of organic compounds [33]. E_LUMO_ indicates the ability of organic compounds to accept electrons, while the opposite is true for E_HOMO_. The compound with the maximum E_LUMO_ value is tert-butyl methyl (0.040 eV), and the compound with the minimum value is diquat (- 0.145 eV), which indicates that diquat is more likely to be able to obtain electrons compared to other organic compounds. The Fukui indices (Fukui) are key to describing the order of decomposition of the molecular structure of organic compounds [19,34]. It includes the electrophilic Fukui indices, the nucleophilic Fukui indices, and the pro-radical Fukui indices [35]. Among them, Fukui(-)_max_ has a maximum of 0.471 e (bromodichloromethane) and a minimum of 0.087 e (diquat). The descriptor bond level reflects the stability of the chemical bonds within a compound molecule. The maximum value of Bond orders(C-C)_min_ is −0.658 (1,3-dichloropropane) and the minimum value is −1.61 (dinoseb). Combined with the correlation plot Fig 1, it can be seen that Bond orders(C-C)_min_, Fukui(-)_max_, E_HUMO_ have a significant correlation with the adsorption effect, which indicates that bond energy, active site, charge distribution, and orbital energy have a strong influence on the adsorption effect of activated carbon.

### 3.3 Correlation analysis of experimental results with structural parameters

Fig 1 shows the correlation between K, 1/n and 31 parameters. It can be seen that there is consistency in the correlation between these two response values (K, 1/n) and the structural parameters, which also indirectly indicates that there is a certain correlation relationship between K and 1/n in the Freundlich adsorption isotherm. According to the absolute value of the correlation coefficient, it can be seen that Bond orders(C-C)min (0.694), Fukui(-)max (0.611), ∑q(O + N) (0.532), and Fukui(-)min (0.515) showed a significant positive correlation with the response values 1/n, while ∑q(H) (−0.598), EHUMO (−0.463), q(C-)max (−0.444), q(CH+)max (−0.414), ∑q(-)/NC (−0.401) showed significant negative correlation with the response values 1/n. A similar correspondence exists for the response value K. The most significant correlation was found between the response values (K, 1/n) and Bond order(C-C)_min_ with a correlation coefficient of 0.694. This structural parameter of Bond order(C-C)_min_ represents the minimum value of the bond orders among the carbon-carbon bonds of a substance, and the magnitude of the value represents the stability of the compound molecule. Another structural parameter is the nucleophilic index (Fukui(-)_max_), which reveals the possible active sites of the reaction. This result indicates that the response values (K, 1/n) are closely related to the active sites. In conclusion, based on the results of the current correlation analysis, it is clear that Bond orders(C-C)_min_ and Fukui(-)_max_ are the main factors affecting the adsorption of activated carbon.

### 3.4 QSAR model construction

The 47 organic compounds studied in this paper were randomly divided into a training set of 38 and a test set of 9 in a ratio of 4:1. Among them, 1,2,3-trichloropropane, isophorone, styrene, cis-1,2-dichloroethene, 1,2-dibromoethane, simazine, atrazine, Alachlor, Metolachlor were selected as test sets for the QSAR model with K as the response value, while tert-butyl methyl ether, benzene, bromobenzene, 1,2-dibromoethane, dibromochloroethane, cis-1,2-dichloroethene, and lindane, picloram, Alachlor were selected as test sets for the model with K as the response value. In this study, the optimal model with low multicollinearity and a good correlation with the response values was identified based on the results obtained from the Qsarins software.

Table 2 presents the results of the QSAR models with K as response values using the stepwise method of Qsarins. From Table 2, the gradual introduction of the number of independent variables, the R, R^2^, and R^2^_adj_ of models 1–5 gradually increase, the SD values gradually become smaller, the F values gradually become larger, and the sig. (significance, see “S3 Table. Meaning of evaluation indexes”, which means the significance values of these five models) are all 0.00. Generally, the determine coefficient R^2^ of the model with a good fit is at least greater than 0.6 [36]. Based on this, models 1 and 2 can be excluded. It is also required that the internal and external validation coefficients q^2^ and Q_ext_^2^ of the optimal model are both greater than 0.5 [37]. It is clear that models 3, 4, and 5 meet the above requirements, but compared to the rest of the models, R2 (0.801) and R2adj (0.770) of model 5 are higher, q2 (0.722) and Qext2 (0.699) of model 5 are higher, the standardized estimation error (SD = 13.332) and the root mean square error (RMSEtr = 12.171) of model 5 are minimized. The model 5 has a lower LOF, which indicates that the model has a good fit with these descriptors. The sig. results indicate that the model is statistically significant. The cross-validation coefficients of the internal verification (Q_LOO_^2 ^= 0.699) for internal validation [38], and the external validation coefficient (R_ext_^2^ = 0.698) for assessing the external prediction ability meet the criterion of greater than 0.5, which indicates that the predictions from the test set are reliable. In addition, the models built from the training set all have small errors for RMSEtr (12.171), MAEtr (8.693), RSStr (5532.415), which effectively evaluates the external predictive power of the model. Therefore, model 5 was determined to be the optimal model for the K model, which contains five variables, namely ∑q(O + N), q(CH+)_max_, Fukui(-)_max_, E_LUMO_, Wiberg(C-C)_min_.

**Table 2:** Results of the K-model organized by the Qsarins.

model	Variables of models	R^2^	R_adj_^2^	R^2^-R_adj_^2^	Sig.	LOF	RMSE_tr_	MAE_tr_	RSS_tr_	SD	F	Q_LOO_^2^	R_ext_^2^	q^2^	Q_ext_^2^
1	∑q(O + N)	0.467	0.451	0.016	4*10^−6^	400.528	20.013	13.329	14419.003	20.59	29.77	0.395	0.345	0.399	0.345
2	∑q(O + N), q(CH+)_max_	0.532	0.503	0.028	4*10^−6^	351.846	18.757	13.454	12666.441	19.59	18.73	0.426	0.598	0.428	0.598
3	∑q(O + N), q(CH+)_max_, E_LUMO_	0.684	0.654	0.030	0	237.384	15.407	10.925	8545.805	16.34	20.70	0.553	0.625	0.557	0.625
4	∑q(O + N), q(CH+)_max_, E_LUMO_, Fukui(-)_max_	0.728	0.693	0.035	0	266.749	14.305	10.532	7366.427	15.41	23.09	0.601	0.645	0.682	0.645
5	∑q(O + N), q(CH+)_max_, E_LUMO_, Fukui(-)_max,_ Wiberg(C-C)_min_	0.801	0.770	0.033	0	148.123	12.171	8.693	5332.415	13.33	24.43	0.699	0.698	0.722	0.699

Table 3 presents the results of the QSAR models with 1/n as response values. From Table 5, with the gradual introduction of the number of independent variables, the R, R^2^, and R^2^_adj_ of models 1–7 gradually increase, the SD value gradually becomes smaller, the F value gradually becomes larger, and the sig. The value of these five models is 0.00. According to the standard requirements of the optimal model, models 1–4 can be excluded. The comparison of the internal and external validation coefficients found that models 5–7 conformed to the criterion of being greater than 0.5. Q_ext_^2^ of model 5 is larger (0.738), and q^2^ of model 7 is larger (0.873). But the LOF of model 5 and model 7 are consistent, and other indicators (K_xx_, MSE_tr_, MAE_tr_, and RSS_tr_) are not showing much difference between the two models. Model 5 has the largest R_ext_^2^ (0.738) and the smallest Q_LOO_^2^ (0.839) in contrast to model 7, which meets the criterion and indicates that the predictions of the test set under model 5 are reliable. Overall, there is not much difference between model 5 and model 7. To avoid overfitting and ensure the prediction performance, model 5 with fewer parameters and simple structure is determined to be the 1/n optimal model following the principle of consistency with the variables of the K model. The model contains Bond orders(C-C)_min_, Bond orders(C-C)_max_, q(C-)_max_, ∑q(-)/N_C_, Wiberg(C-C)_min_.

**Table 3:** Results of the 1/n-model organized by the Qsarins.

model	Variables of models	R^2^	R_adj_^2^	R^2^-R_adj_^2^	Sig.	LOF	RMSE_tr_	MAE_tr_	RSS_tr_	SD	F	Q_LOO_^2^	R_ext_^2^	q^2^	Q_ext_^2^
1	Bond orders(C-C)_min_	0.719	0.711	0.008	4*10^−6^	0.008	0.087	0.072	0.285	0.089	91.951	0.688	0.001	0.689	0.001
2	Bond orders(C-C)_min_, Bond orders(C-C)_max_,	0.765	0.751	0.014	1*10^−6^	–	–	–	–	–	–	–	–	0.731	0.180
3	Bond orders(C-C)_min_, Bond orders(C-C)_max_, ∑q(-)/N_C_,	0.810	0.794	0.016	1*10^−6^	–	–	–	–	–	–	–	–	0.767	0.450
4	Bond orders(C-C)_min_, Bond orders(C-C)_max_, ∑q(-)/N_C_, Wiberg(C-C)_min_,	0.846	0.827	0.019	0	–	–	–	–	–	–	–	–	0.802	0.485
5	Bond orders(C-C)_min_, Bond orders(C-C)_max_, ∑q(-)/N_C_, Wiberg(C-C)_min_, q(C-)_max_	0.885	0.867	0.018	0	0.006	0.055	0.047	0.116	0.060	49.406	0.839	0.738	0.840	0.738
6	Bond orders(C-C)_min_, Bond orders(C-C)_max_, ∑q(-)/N_C_, Wiberg(C-C)_min_, q(C-)_max_, Fukui(+)_max_,	0.904	0.885	0.019	0.	0.006	0.051	0.043	0.098	0.056	48.567	0.860	0.734	0.861	0.740
7	Bond orders(C-C)_min_, Bond orders(C-C)_max_, ∑q(-)/N_C_, Wiberg(C-C)_min_, q(C-)_max_, Fukui(+)_max_, Fukui(+)_min_	0.917	0.897	0.019	1*10^−6^	0.006	0.048	0.039	0.085	0.053	47.166	0.873	0.704	0.873	0.704

**Fig 2:**
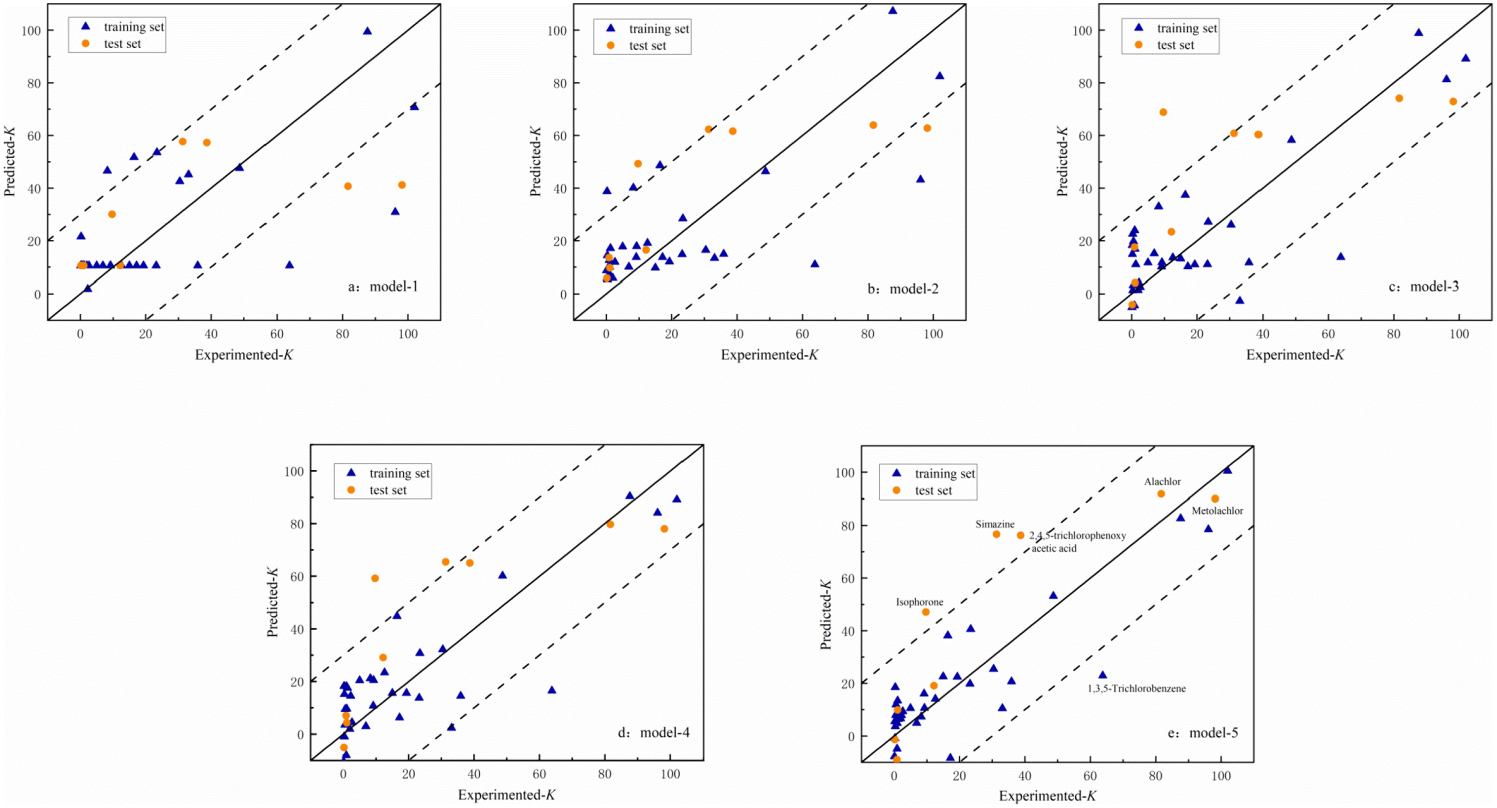
Scatter plot of predicted versus experimental values for models about K; a) model-1; b) model-2; c) model-3; d) model-4; e) model-5.

**Fig 3:**
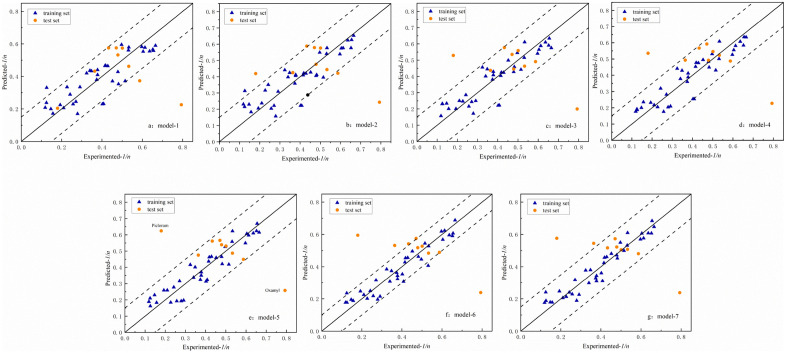
Scatter plot of predicted versus experimental values for models about 1/n: a) model-1; b) model-2; c) model-3; d) model-4; e) model-5; f) model-6; g) model-7.

In addition, we can identify potential outliers by the plot of predicted versus experimental response values (Figs 2 and 3). Fig 2 shows the scatter distributions of predicted and experimental values of the K models on the training and test sets. It can be observed that model 5 has a better fit and a denser distribution compared to the other models. Most of the predicted data are distributed around the 1:1 (y = x) regression line, only three compounds such as simazine, 2,4,5-trichlorophenoxy acetic acid, and isophorone are outside the range of variation of the residuals. In the scatter plot of the 1/n model, only two compounds (picloram and nematicarb) are slightly further apart from the regression line.

Further analysis showed that the predictive ability of the model built for these structures was limited due to triazines (atrazine and simazine), and carbamate pesticides containing a nitrogen heterocyclic ring (oxamyl), which were underrepresented in the training set, thus leading to a limited predictive ability of the model built for these structures. Therefore, we have already excluded these categories when establishing the K model. The adsorption mechanisms of 2,4,5-trichlorophenoxy acetic acid, isophorone, simazine and picloram may involve hydrophobic interactions, and the present study focused on analyzing parameters such as active site reactivity and electronic effects, thus leading to a large bias in model predictions.

The adsorption models studied by previous authors are listed in Table 4. Most of them focused on the exploration of the hydrophobic properties for a single type of substance, and the parameters chosen were different from those of the present study, which made the compounds with large deviations in the present study.

**Table 4:** Modeling studies in the literature similar to this study.

Research literature (year)	Methods	Type of datasets	Key impact factors
Blum et al. (1994) [8]	MLR	aromatic and aliphatic	molecular connectivity indices
De Ridder et al. (2010) [5]	MLR	organic micropollutants	hydrophobicity (log D), polarizability
Gong et al. (2015) [39]	PLS	mono-nitro aromatic compounds	molecular orbital (E_LUMO_), the atomic net charges (Q_C_^-^)
Zhao et al.(2018) [40]	MLR	cationic pharmaceuticals	octanol-water partition coefficient (log P), polar surface area, molecular weight (MW)

Multiple linear regression (MLR), partial-least-squares regression (PLS)

## 4 Discussion

### 4.1 Mechanism explanation

Each quantum chemical parameter in the model plays an important role in the interpretation of the optimal model, and the combined analysis of all descriptors helps to reveal the rules of absorption. The above two models involve different independent variables. For K and 1/n in the Freundlich adsorption equation, they cannot completely express the adsorption properties of the adsorbent for the organic compounds independently [41,42]. Hence, two adsorption models based on the Freundlich equation are considered, which is essential to understanding the adsorption mechanism [19,43].

Both the K optimal model and the 1/n optimal model contain Wiberg(C-C)_min_, and this parameter is used to measure the strength of covalent bonds between carbon and carbon atoms in a molecule. A lower value means that the carbon-carbon bonds in the molecule are weaker, and its structure may be less stable. As can be seen from the regression coefficients in the model, this descriptor is negatively correlated with K and positively correlated with 1/n. From this we can infer that the lower Wiberg(C-C)_min_ of the compound, the more unstable the structure of the molecule is, which may have an effect on the adsorption between the compound molecules and activated carbon. Combined with S2 Table, it can be verified from metolachlor (1.103), cyanazine (1.233), and alachlor (1.104) with the smaller Wiberg (C-C) _min_. Their corresponding K values (98200, 102000, and 81,700) are higher than the average value (21201). Correspondingly, the corresponding n values (0.125, 0.126, 0.257) are also smaller than the mean value (0.407).

It is noteworthy that in the correlation analysis, K and 1/n both showed a low correlation coefficient with Wiberg(C-C)min (−0.033 and −0.059). Similarly ELUMO (−0.104 and 0.149) and Bond orders(C-C)max (0.306 and −0.291) are also low. In the K-optimal model, the value of the corresponding coefficient for ELUMO is large (−391.288), but the correlation with K in the correlation analysis is small (−0.104). The reason for this aspect may be the complementary effect between the two parameters, where the molecules with lower E_LUMO_ may react with the activated carbon surface through electron transfer, while the weaker Wiberg(C-C)_min_ indicates a decrease in the stability of the bonds in the molecule, which releases the active fragments to enhance adsorption. Thus the synergy between the two explains the co-regulation of the adsorption capacity by electronic effects and structural stability. The synergistic effect between ELUMO and Wiberg(C–C)min refers to their complementary roles in enhancing physisorption. A lower ELUMO value indicates a greater electron-accepting ability, facilitating π–π interactions or weak charge-transfer with the electron-rich graphene layers of activated carbon. Concurrently, a smaller Wiberg(C–C)min reflects weaker intrinsic carbon-carbon bonds within the adsorbate molecule, which may allow for better geometric adaptation and dispersion interactions with the heterogeneous carbon surface. This combined electronic and structural effect optimizes the adsorption affinity, a phenomenon consistent with studies on the adsorption of aromatic and polar compounds onto carbonaceous materials In the 1/n-optimal model, the corresponding coefficients for Bond orders(C-C)_max_ are negative (−2.775), while those for Wiberg(C-C)_min_ are positive (0.037). Higher Bond orders(C-C)_max_ may inhibit molecular conformational change and limit its ability to fit into the activated carbon pores, and lower Wiberg(C-C)_min_ may promote localized bond breaking or distortion and enhance molecular interactions with adsorption sites. Thus these two parameters regulate the structural dynamics of the molecules during adsorption, thereby affecting the degree of nonlinearity of the isotherms. We may also illustrate this using example compounds. Aldicarb (ELUMO = –0.032 eV, K = 8,270): Its relatively low ELUMO favors electron acceptance from carbon’s π-system, enhancing adsorption via charge transfer; Oxamyl (ELUMO = –0.048 eV, K = 1,740): Its higher (less negative) ELUMO indicates poorer electron affinity, reducing charge-transfer potential and adsorption—consistent with its lower K value. In conclusion, analysis of individual variables cannot capture this synergistic effect. Moreover, in subsequent K-model analyses, the Wiberg(C-C)min enhanced robustness (q^2^: 0.682 → 0.722; SD: 14.305 → 12.171). Within the 1/n model, it refined the nonlinear prediction (R^2^: 0.885 → 0.917). Its VIF values (K-type: 1.173; 1/n-type: 1.369; Tables 5-6) confirmed the absence of multicollinearity, justifying its retention.

**Table 5:** Statistical values of variables for the optimal K-model.

Variable	Regression coefficients	VIF	t	Sig.
∑q(O + N)	−125.215	3.675	−10.982	0.000
q(CH+)_max_	−695.036	4.138	−7.413	0.000
E_LUMO_	−391.288	1.531	−5.093	0.000
Fukui(-)_max_	−98.670	1.248	−3.867	0.000
Wiberg(C-C)_min_	−6.771	1.173	−3.455	0.001
Criteria	–	1-5	>2.179	<0.05

**Table 6:** Statistical values of variables for the optimal 1/n-model.

Variable	Regression coefficients	VIF	t	Sig.
Bond orders(C-C)_min_	0.305	2.758	5.821	0.000
Bond orders(C-C)_max_	−2.775	2.305	−6.382	0.000
∑q(-)/N_C_	−3.182	3.512	−5.313	0.000
Wiberg(C-C)_min_	0.037	1.369	4.072	0.000
q(C-)_max_	0.717	2.734	3.312	0.002
Criteria	–	1-5	>2.179	<0.05

The electrophilic indices (Fukui(-)_max_) mainly refers to the active site where the compound is most susceptible to electrophilic reaction, and the active site is the main factor affecting the adsorption effect [44,45]. In the present study, this parameter is inversely proportional to the K value in this model, which means that with higher Fukui(-)_max_ organic compounds have a poor adsorption effect. We can infer that the higher the Fukui(-) _max_ value of the compound, it means that the compound is easy to lose electrons. This may lead to the formation of transient adsorption complexes or quasi-stable adsorption states (e.g., through electrostatic or π-π interactions) during the physisorption process, which could be less favorable for stable adsorption, thereby reducing the overall adsorption performance. For example, the Fukui(-)_max_ of 1,2-dichloroethane (0.3565) was higher than that of 1,2,3-trichloropropane (0.2651), and the K showed that 1,2,3-trichloropropane had a better adsorption capacity.

Bond orders(C-C)_min_ and Bond orders(C-C)_max_ reflect the stability of the molecule. The 1/n-model shows that both are negatively and positively correlated with 1/n, respectively, which reflects that the stability of the chemical bonds influences the degree of nonlinearity of the compounds in the adsorption process. In the adsorption process, smaller Bond orders (C-C) imply weaker carbon-carbon bonds, which makes the compounds easier to be adsorbed by the activated carbon, the closer the adsorption process is to linearity.

According to the correlation (Fig 1), ∑q(O + N) is much more correlated with K than ∑q(O) and ∑q(N), which indicates that the overall charge distribution of the compound molecules has a greater influence on the adsorption effect of activated carbon. For example, diquat has the largest value of ∑q(O + N) (0.1538), while K (2260) is much smaller than the average level; glyphosate has the smallest (−1.5546), while K (87600) is much larger than the average level. ∑q(-)/N_C_ reflects the overall reflection of the negative charge of the organic compound, and the larger its value, the stronger the overall electronegativity of the compound and the more difficult it is to be adsorbed. This point also explains the relationship that ∑q(-)/N_C_ is negatively correlated with 1/n. The q(CH+)_max_ and q(C-)_max_ reflect the charge distribution of different atoms in organic compounds and molecular polarity [46], which affect the adsorption performance of the adsorbent on the compounds, and hence the linearity of the adsorption isotherm.

Although hydrophobicity parameters (e.g., logP) were not directly introduced in this study, the charge distribution parameters included in the model may also indirectly reflect the association between molecular polarity and hydrophobicity. Combined with S2 Table, it can be found that it may not be possible to accurately assess the adsorption performance of compounds based on the magnitude of a single parameter, while K and n together describe the overall adsorption behavior of the adsorbent on compounds in Freundlich, and therefore the explanation of the adsorption mechanism of activated carbon is valuable only when the K and 1/n are considered together with structure parameters.

In this study, it was found that for compounds containing halogen, nitrogen or oxygen functional groups (e.g., pesticides, pharmaceutical intermediates), the adsorption behavior is more significantly affected by the reactivity of the active site and the electronic effect, although the hydrophobic interaction is crucial for the adsorption mechanism. In the developed model, Fukui(-)_max_ represents the easily attacked active site of the compound, which is capable of directly interacting chemically with the functional groups on the surface of the activated carbon; E_LUMO_, Bond orders(C-C)_min_ and Bond orders(C-C)_min_ represent the ability to accept electrons and break chemical bonds, which may affect the charge transfer between adsorbent and compound. The two optimal models developed contain the Wiberg(C-C)_min_, which reflect the electron density distribution of the bonds and the bond strengths. This finding suggests that the relative importance of reactivity and hydrophobicity parameters may vary with molecular structure differences in complex pollutant systems. The modeling by quantum chemical parameters in this study provides a new perspective for understanding the reactivity-dominated adsorption mechanism.

Take the example of aldicarb and oxamyl. Fukui(-)_max_ corresponds to the sites in its molecule that are easily attacked by oxygen-containing functional groups on the surface of the activated carbon, and higher values allow it to bind to the surface of the activated carbon through hydrogen bonding or electrostatic interaction, enhancing the adsorption capacity. The lower E_LUMO_ indicates that the activated carbon surface may transfer electrons to aldicarb through charge transfer to form an adsorption complex. The magnitude of the parameters of Bond orders(C-C)_min_ and Wiberg(C-C)_min_ is mainly reflected in the C-N bonding in the methylcarbamate group in aldicarb, which is a weak bonding that makes aldicarb susceptible to exchange electrons with the activated carbon surface. For oxamyl, its low Fukui(-)_max_ makes it difficult to form chemical or hydrogen bonds with functional groups on the surface of activated carbon. Higher E_LUMO_ implies low charge transfer efficiency between it and activated carbon, making it difficult to form stable adsorption complexes. Higher Bond orders(C-C)_min_ and Wiberg(C-C)_min_ indicate greater chemical bond strength and more stable electron density distribution in the oxamyl molecules, resulting in a lower possibility of electron exchange or bonding with the activated carbon surface.

### 4.2 Model validation

To check the stability of the optimal model, t-test and VIF test were conducted for the model, respectively. The statistical values of the independent variables regarding the K optimal model and the 1/n optimal model are shown in Tables 5 and 6.

The results show that the values of the sig. of all the respective independent variables in the K optimal model and the 1/n optimal model are less than 0.05, and the absolute value of t is greater than 2.179. For the K optimal model, the order of magnitude of the absolute values of t is ∑q(O + N) > q(CH+)max > ELUMO > Fukui(-)max > Wiberg(C-C)min, and for the 1/n-optimization model, it is Bond orders(C-C)min > Bond orders(C-C)max > ∑q(-)/NC > Wiberg(C-C)min > q(C-)max, which indicates that these variables are acceptable. The VIF is used to test for multicollinearity among the independent variables of a model [47]. The criteria for this indicator require that if VIF > 10, the model equation is unreliable and needs to be retested or re-modeled; if VIF = 1–5, the model equation is acceptable; and if VIF = 1, there is no correlation between every variable of the model. Tables 5 and 6 show that all the VIF values in the optimal model meet the criteria of 1–5. In addition, the external validation coefficients (K-model: Rext2 = 0.699, Qext2 = 0.668; 1/n-model: R_ext_^2^ = 0.738, Q_ext_^2^ = 0.738) of the model all meet the criterion of being greater than 0.5, which suggests that the model has a good predictive potential.

### 4.3 Y-randomization verification and LMO cross-validation

To check the robustness of the model, Y-randomized validation [48] and Leave-Many-Out cross-validation were performed on the developed model.

**Fig 4:**
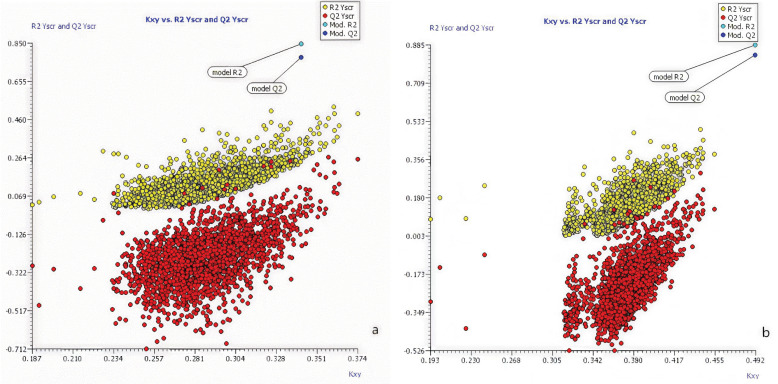
Comparison of Y-randomized model with the optimal model: a) the optimal model for K; b) the optimal model for 1/n.

**Fig 5:**
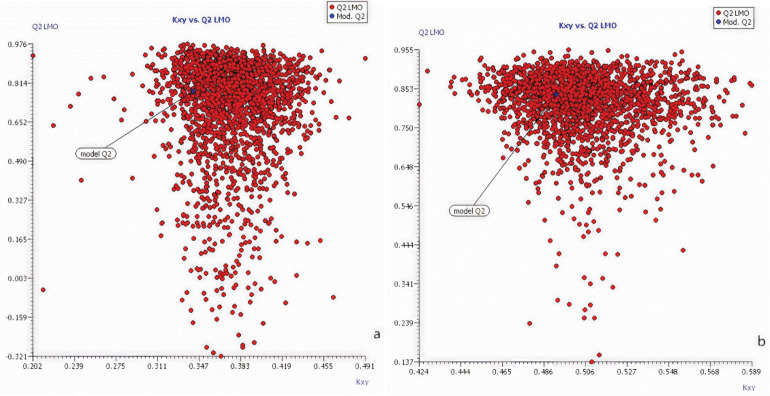
Comparison of the LMO model with the optimal model: a) the optimal model for K; b) the optimal model for 1/n.

For a stable QSAR model, R^2^_yand_ and Q^2^_yrand_ of the Y-randomized model should be lower compared to the original model’s R^2^ and Q^2^, otherwise, it means that the original model is unreliable. The results are shown in Fig 4, which determined that the two optimal models established in this study have better robustness and stability. The internal prediction ability of the model is also determined based on the comparison of the new cross-validation coefficient (Q_LMO_^2^) with the Q_LMO_^2^ of the original model. The results are shown in Fig 5, the performance of the new LMO model is basically the same as the original model (the optimal model established), so the above two models meet the requirements and passed the validation.

### 4.4 Areas of applicability

**Fig 6:**
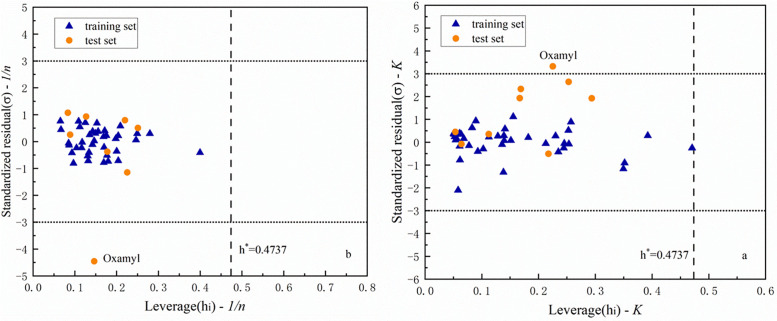
Williams plot of the optimal model: a) the optimal model for K; b) the optimal model for 1/n.

To make the models constructed in this study better applied to the study of the adsorption performance of other organic compounds, we also used Williams plots to observe the applicability domains (APDs) of the two optimal models constructed. The details are shown in Fig 6.

The domain of the application test mainly illustrates the range of applicability predicted by the optimal model [49], and only the predictions of the compounds belonging to this field of study are considered to be reliable [50]. The constructed models can predict when the predicted organic compounds satisfy the standard residuals (σ) between −3, 3 and the leverage value ($h_{i}$) is below the warning bar ($h^{*}$) [29,51]. As shown in Fig 6, most of the standard residuals of the organic compounds satisfy the range. However, it was found that the oxamyl in the test set was not in the APD and exceeded the critical value in the K-williams and 1/n-williams plots. The analysis shows that oxamyl is a carbamate compound with a nitrogen-containing heterocyclic structure, which is significantly distinct from the others in structure. This difference may cause its experimental results to be different from the predicted results. In general, the two optimal models still have better stability and prediction ability.

In addition, in order to quantitatively define the model boundaries, we performed Tanimoto similarity analysis based on molecular fingerprints.

**Fig 7:**
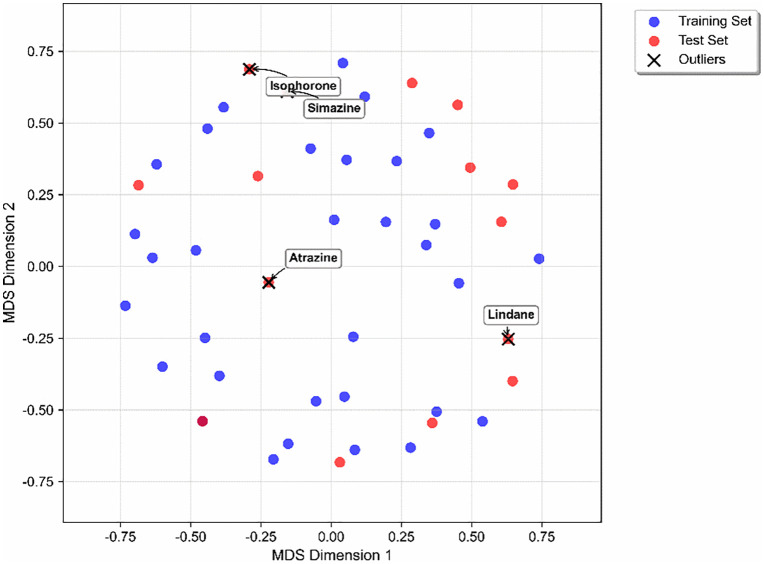
Chemical space distribution based on tanimoto similarity.

As shown in the chemical-space distribution derived from molecular-fingerprint Tanimoto similarities (Fig 7), the MDS plot reveals a relatively well-defined and cohesive chemical space occupied by the majority of the training set compounds, indicating structural consistency within the model’s training domain. The test set compounds are generally intermingled with or situated on the periphery of the training set cloud, suggesting that the external validation set shares a reasonable degree of structural similarity with the training compounds, which is crucial for reliable predictive modeling.

### 4.5 Comparison with models in the literature

According to Table 4, the existing adsorption QSAR models mainly focus on the hydrophobic properties of organic compounds. Only one type of compound is included in the research object, so the developed model may only be applicable to this specific type of compound, and it is difficult to be extended to other types of substances. In addition, the lack of sufficient model performance verification methods may cause problems with the reliability and applicability of the model. This study involved a variety of compounds (including halogenated hydrocarbons, Monoaromatic Hydrocarbons and nitrogen-/oxygen-containing organics), thereby enhancing the applicability of the model.

The second aspect is that time-consuming experiments are normally needed to obtain the exact hydrophobic property of each organic compound, especially for the emerging organics. Actually, the hydrophobic property of each organic compound should also be decided by the molecular structure feature which could normally be quantified through the quantum parameters of the compound. Therefore, another advantage of the QSAR model is the direct connection of organic adsorption characteristics with the molecular structures through the quantum parameters which can be calculated though computer without tedious physical chemistry experiments, especially for the emerging organic compounds.

Furthermore, this model was developed using adsorption data exclusively from Fitrasorb-400 activated carbon, which serves as a well-characterized reference material. The identified QSARs capture the fundamental influence of adsorbate properties (electronic and structural) on adsorption affinity. While the absolute adsorption capacity (K value) can be influenced by the surface heterogeneity of different activated carbons (e.g., oxygen-containing functional groups, ash content), the underlying trends described by the quantum chemical descriptors are expected to be qualitatively transferable to carbons with similar non-specific surface domains. To quantitatively predict adsorption onto carbons with vastly different surface chemistries, future extensions of this framework will incorporate material-specific descriptors (e.g., surface O/C ratio, point of zero charge) into a comprehensive model.

### 4.6 Limitations and future perspectives

It is important to note the limitations of the current model. The experimental data were obtained under natural aqueous conditions (pH 5.3–8.0) without adjustment, and the descriptors were calculated for neutral molecular species. Consequently, the model’s predictive ability for strongly ionizable emerging contaminants (e.g., metalaxyl and simazine) under different pH conditions may be limited. Future work will focus on integrating pH-corrected molecular descriptors to significantly expand the applicability domain of the QSAR model.

## 5 Conclusion

The modeling results showed that the two key parameters (K and 1/n) of the Freundlich Isotherm are correlated well with certain quantum chemical parameters which indicates that the applicability of the empirical Isotherm is actually related to the molecular structure characteristics of the organic compounds. The specific quantum parameters determining the value of K were discovered as ∑q(O + N), q(CH+)_max_, E_LUMO_, Fukui(-)_max_, Wiberg(C-C)_min_, which indicates that the charge distribution, carbon bond energy and active site energy in the molecular structure are the main factors affecting the organic adsorption efficiency by activated carbon. The QSAR model of 1/n exhibited the similar finding that 1/n value is also correlated with the molecular structure characteristics.

The two optimal models were further confirmed to be stable, robust, and accurate by the standard evaluation. The two QSAR models can be used to predict the adsorption efficiency of other similar compounds (subject to model applicability domain testing) based on the related quantum parameters. Therefore, this study provides an important and convenient reference method for evaluating the applicability of activated carbon adsorption unit to control various emerging organic pollutants in drinking water plants and also provides them a basis for constructing an intelligence management system.

Future work will extend the QSAR framework to multi-solute systems by incorporating competitive adsorption coefficients and molecular dynamics simulations.

## Supporting information

S1 Table Meaning of parametric descriptors of compounds. (DOCX)

S2 Table Information on Freundlich parameters and 31 molecular parameters for 47 organic compounds. (DOCX)

S3 Table Meaning of evaluation indexes. (DOCX)
