# Supplementary material for: Quantitative structure activity relationship (QSAR) modeling for adsorption of organic compounds by activated carbon based on Freundlich adsorption isotherm
Source: PLoS One. 2025 Dec 15;20(12):e0338483. doi: 10.1371/journal.pone.0338483 (PMC12704836; doi:10.1371/journal.pone.0338483)
Supplement: S1 Table — (DOCX) [file pone.0338483.s001.docx]

***Supporting information***

**S1Table. Meaning of parametric descriptors of compounds**

| **Descriptors** | **Connotation** | **Unit** |
| --- | --- | --- |
| μ | Dipole moment in vacuum | Debye |
| q(CH+) _max_ | Maximum value of the positive partial charge of a hydrogen atom connected to a carbon atom | e |
| q(CH+) _min_ | Minimum value of the positive partial charge of a hydrogen atom connected to a carbon atom | e |
| q(C-)_max_ | Maximum value of partial charge on a carbon atom | e |
| q(C-)_min_ | Minimum value of partial charge on a carbon atom | e |
| E_(B3LYP)_ | Total molecular energy under B3LYP calculations | kcal/mol |
| Fukui (+) _max_ | Maximum value of nucleophilic Fukui index in carbon atom | e |
| Fukui (+) _min_ | Minimum value of nucleophilic Fukui index in carbon atom | e |
| Fukui (-) _max_ | Maximum value of electrophilic Fukui index in carbon atom | e |
| Fukui (-) _min_ | Minimum value of electrophilic Fukui index in carbon atom | e |
| Fukui (0) _max_ | Maximum value of free radical affinity Fukui index in carbon atoms | e |
| Fukui (0) _min_ | Minimum value of free radical affinity Fukui index in carbon atoms | e |
| E_HOMO_ | Highest energy of an occupied molecular orbital | eV |
| E_LUMO_ | Lowest energy of unoccupied molecular orbital | eV |
| E_GAP_ | Difference between E_LUMO_ and E_HOMO_ | eV |
| Bond orders(C-C) _max_ | C-C bond level maximum value | **-** |
| Bond orders(C-C) _min_ | C-C bond level minimum value | **-** |
| Bond orders(C-H) _max_ | C-H bond level maximum | **-** |
| Bond orders(C-H) _min_ | C-H bond level minimum | **-** |
| Wiberg(C-C) _max_ | Maximum value of the Wiberg index for the strongest bond between any two carbon atoms in a molecule | **-** |
| Wiberg(C-C) _min_ | Minimum value of the Wiberg index for the weakest bond between any two carbon atoms in a molecule | **-** |
| Wiberg(C-H) _max_ | Maximum value of the Wiberg index for the strongest bond between any carbon atom and hydrogen atom in the molecule | **-** |
| Wiberg(C-H) _min_ | Minimum value of the Wiberg index for the weakest bond between any carbon atom and hydrogen atom in the molecule | **-** |
| ∑q(O) | Total charge of all oxygen atoms in the molecule | e |
| ∑q(N) | Total charge of all nitrogen atoms in the molecule | e |
| ∑q(O+N) | Total charge of all nitrogen and oxygen atoms in the molecule | e |
| ∑q(C) | Total charge of all carbon in the molecule | e |
| ∑q(H) | Total charge of all hydrogen in the molecule | e |
| ∑q (H)/N_H_ | The average charge shared by a single hydrogen atom in a molecule | - |
| ∑q (-)/N_C_ | The average negative charge shared by a single carbon atom in a molecule | - |
| MW | relative molecular mass | g/mol |
