# Supplementary material for: Quantitative structure activity relationship (QSAR) modeling for adsorption of organic compounds by activated carbon based on Freundlich adsorption isotherm
Source: PLoS One. 2025 Dec 15;20(12):e0338483. doi: 10.1371/journal.pone.0338483 (PMC12704836; doi:10.1371/journal.pone.0338483)
Supplement: S2 Table — (DOCX) [file pone.0338483.s002.docx]

***Supporting information***

**S2 Table. Information on Freundlich parameters and 31 molecular parameters for 47 organic compounds (part 1)**

| **Name** | **Molecular formula** | **cas** | **K（×10^3^）** | **1/n** | **μ** | **q(CH+)_max_** | **q(CH+)_min_** | **q(C-)_max_** | **q(C-)_min_** | **E_(B3LYP)_** |
| --- | --- | --- | --- | --- | --- | --- | --- | --- | --- | --- |
| 1,2-Dichloroethane | C_2_H_4_Cl_2_ | 107-06-2 | 0.129 | 0.533 | 3.952 | 0.066 | 0.056 | 0.009 | -0.130 | -999.117 |
| Benzene | C_6_H_6_ | 64268-28-6 | 1.26 | 0.533 | 0.000 | 0.045 | 0.045 | -0.045 | -0.045 | -232.333 |
| Lindane | C_6_H_6_Cl_6_ | 58-89-9 | 15 | 0.433 | 3.120 | 0.063 | 0.045 | 0.025 | -0.082 | -2993.735 |
| Toluene | C_7_H_8_ | 108-88-3 | 5.01 | 0.429 | 0.571 | 0.044 | 0.038 | 0.003 | -0.081 | -271.666 |
| 2,4,5-trichlorophenoxy acetic acid | C_9_H_7_Cl_3_O_3_ | 1928-37-6 | 43 | 0.210 | 5.183 | 0.063 | 0.050 | 0.213 | -0.055 | -1953.723 |
| Alachlor | C_14_H_20_ClNO_2_ | 15972-60-8 | 81.7 | 0.257 | 7.575 | 0.058 | 0.024 | 0.165 | -0.117 | -1211.261 |
| Atrazine | C_8_H_14_ClN_5_ | 102029-43-6 | 38.7 | 0.291 | 7.080 | 0.132 | 0.027 | 0.153 | -0.103 | -1047.541 |
| Carbofuran | C_12_H_15_NO_3_ | 1563-66-2 | 16.4 | 0.408 | 2.768 | 0.141 | 0.033 | 0.213 | -0.090 | -747.038 |
| Dicamba | C_8_H_6_Cl_2_O_3_ | 1918-00-9 | 33.1 | 0.147 | 1.319 | 0.198 | 0.034 | 0.208 | -0.042 | -1454.781 |
| Glyphosate | C_3_H_8_NO_5_P | 1071-83-6 | 87.6 | 0.119 | 4.258 | 0.198 | 0.048 | 0.219 | -0.038 | -891.699 |
| Metolachlor | C_15_H_22_ClNO_2_ | 51218-45-2 | 98.2 | 0.125 | 7.660 | 0.063 | 0.020 | 0.160 | -0.111 | -1250.590 |
| Simazine | C_7_H_12_ClN_5_ | 122-34-9 | 31.3 | 0.227 | 6.987 | 0.133 | 0.035 | 0.150 | -0.102 | -1008.208 |
| Bromoform | CHBr_3_ | 2909-52-6 | 0.929 | 0.665 | 1.352 | 0.069 | 0.069 | 0.034 | 0.034 | -7761.158 |
| 1,1,1-Trichloroethane | C_2_H_3_Cl_3_ | 71-55-6 | 0.335 | 0.531 | 2.557 | 0.052 | 0.052 | 0.127 | -0.088 | -1458.730 |
| Bromodichloromethane | CHBrCl_2_ | 75-27-4 | 0.241 | 0.655 | 1.506 | 0.072 | 0.072 | 0.074 | -0.053 | -3533.315 |
| 1,1-Dichloroethane | C_2_H_4_Cl_2_ | 75-34-3 | 0.47 | 0.515 | 1.927 | 0.064 | 0.064 | 0.039 | -0.079 | -997.869 |
| Isophorone | C_9_H_14_O | 78-59-1 | 9.75 | 0.271 | 6.492 | 0.049 | 0.025 | 0.137 | -0.089 | -426.789 |
| 1,2-Dichloropropane | C_3_H_6_Cl_2_ | 78-87-5 | 0.313 | 0.597 | 4.163 | 0.063 | 0.029 | 0.042 | -0.137 | -1038.451 |
| 1,1,2-Trichloroethane | C_2_H_3_Cl_3_ | 25323-89-1 | 0.365 | 0.652 | 3.937 | 0.073 | 0.066 | 0.068 | -0.110 | -1458.736 |
| Trichloroethylene | C_2_HCl_3_ | 79-01-6 | 2 | 0.482 | 1.242 | 0.072 | 0.072 | 0.028 | -0.033 | -1457.490 |
| Diquat | C_12_H_12_N_2_+_2_ | 2764-72-9 | 2.26 | 0.242 | 2.229 | 0.105 | 0.085 | 0.108 | 0.024 | -573.875 |
| Dinoseb | C_10_H_12_N_2_O_5_ | 88-85-7 | 30.4 | 0.279 | 9.480 | 0.180 | 0.026 | 0.109 | -0.085 | -874.047 |
| o-Chlorotoluene | C_7_H_7_Cl | 95-49-8 | 23.2 | 0.378 | 2.132 | 0.051 | 0.036 | 0.014 | -0.084 | -731.294 |
| o-Dichlorobenzene | C_6_H_4_Cl_2_ | 95-50-1 | 19.3 | 0.378 | 3.574 | 0.057 | 0.057 | 0.012 | -0.059 | -1151.583 |
| Dibromochloropropane | C_3_H_5_Br_2_Cl | 96-12-8 | 6.91 | 0.501 | 2.351 | 0.062 | 0.042 | 0.018 | -0.108 | -5725.921 |
| 1,2,3-Trichloropropane | C3H5Cl3 | 96-18-4 | 1.08 | 0.613 | 2.390 | 0.063 | 0.043 | 0.033 | -0.109 | -1498.075 |
| Ethyl benzene | C8H10 | 100-41-4 | 9.27 | 0.415 | 0.589 | 0.043 | 0.028 | 0.003 | -0.083 | -310.995 |
| Styrene | C8H8 | 100-42-5 | 12.2 | 0.479 | 0.249 | 0.047 | 0.040 | -0.010 | -0.086 | -309.762 |
| p-Xylene | C8H10 | 106-42-3 | 12.6 | 0.418 | 0.000 | 0.040 | 0.037 | -0.003 | -0.082 | -310.999 |
| p-Chlorotoluene | C7H7Cl | 106-43-4 | 35.9 | 0.340 | 3.087 | 0.050 | 0.041 | 0.011 | -0.088 | -731.293 |
| 1,2-Dibromoethane | C2H4Br2 | 106-93-4 | 0.888 | 0.471 | 0.000 | 0.054 | 0.054 | -0.011 | -0.011 | -5226.963 |
| 1,3,5-Trichlorobenzene | C6H3Cl3 | 108-70-3 | 63.8 | 0.324 | 0.003 | 0.060 | 0.060 | 0.034 | -0.048 | -1611.209 |
| Bromobenzene | C_6_H_5_Br | 108-86-1 | 17.2 | 0.364 | 2.435 | 0.053 | 0.050 | 0.000 | -0.046 | -2805.881 |
| Chlorobenzene | C_6_H_5_Cl | 108-90-7 | 9.17 | 0.348 | 2.427 | 0.053 | 0.051 | 0.018 | -0.081 | -691.960 |
| 2,4-Dinitrotoluene | C_7_H_6_N_2_O_4_ | 121-14-2 | 96.1 | 0.157 | 7.029 | 0.067 | 0.048 | 0.053 | -0.075 | -680.807 |
| 1,3-Dichloropropane | C_3_H_6_Cl_2_ | 142-28-9 | 0.897 | 0.497 | 3.399 | 0.056 | 0.040 | 0.007 | -0.133 | -1038.451 |
| 1,1-Dichloropropene | C_3_H_4_Cl_2_ | 563-58-6 | 2.67 | 0.374 | 2.792 | 0.058 | 0.033 | 0.022 | -0.073 | -1037.204 |
| 1,1,1,2-Tetrachloroethane | C_2_H_2_Cl_4_ | 630-20-6 | 1.07 | 0.604 | 2.166 | 0.062 | 0.062 | 0.115 | -0.090 | -1918.348 |
| tert-Butyl methyl ether | C_5_H_12_O | 188595-68-8 | 0.218 | 0.479 | 1.985 | 0.039 | 0.028 | 0.090 | -0.092 | -273.085 |
| Picloram | C_6_H_3_Cl_3_N_2_O_2_ | 1918-02-1 | 23.4 | 0.180 | 5.042 | 0.197 | 0.129 | 0.205 | -0.059 | -1871.276 |
| Metribuzin | C_8_H_14_N_4_OS | 21087-64-9 | 48.7 | 0.193 | 2.505 | 0.129 | 0.022 | 0.160 | -0.093 | -1005.919 |
| Cyanazine | C_9_H_13_ClN_6_ | 21725-46-2 | 102 | 0.126 | 6.067 | 0.138 | 0.032 | 0.157 | -0.090 | -1139.807 |
| Dibromochloromethane | CHBr_2_Cl | 93951-99-6 | 0.585 | 0.636 | 1.438 | 0.070 | 0.070 | 0.054 | -0.050 | -5647.236 |
| trans-1,2-Dichloroethylene | C_2_H_2_Cl_2_ | 156-60-5 | 0.618 | 0.452 | 0.000 | 0.068 | 0.068 | -0.016 | -0.052 | -997.871 |
| cis-1,2-Dichloroethylene | C_2_H_2_Cl_2_ | 156-59-2 | 0.202 | 0.587 | 2.553 | 0.072 | 0.072 | -0.017 | -0.055 | -997.873 |
| Aldicarb | C_7_H_14_N_2_O_2_S | 116-06-3 | 8.27 | 0.402 | 4.113 | 0.140 | 0.031 | 0.203 | -0.090 | -933.526 |
| Oxamyl | C_7_H_13_N_3_O_3_S | 23135-22-0 | 1.74 | 0.793 | 8.724 | 0.143 | 0.040 | 0.208 | -0.069 | -1062.940 |

**S2 Table. Information on Freundlich parameters and 31 molecular parameters for 47 organic compounds (part 2)**

| **Name** | **Fukui(-)_max_** | **Fukui(-)_min_** | **Fukui(+)_max_** | **Fukui(+)_min_** | **Fukui(0)_max_** | **Fukui(0)_min_** | **E_LUMO_** | **E_HOMO_** | **E_GAP_** |
| --- | --- | --- | --- | --- | --- | --- | --- | --- | --- |
| 1,2-Dichloroethane | 0.357 | 0.040 | 0.454 | 0.009 | 0.405 | 0.025 | -0.008 | -0.310 | 0.302 |
| Benzene | 0.176 | 0.045 | 0.155 | 0.046 | 0.133 | 0.046 | -0.013 | -0.261 | 0.248 |
| Lindane | 0.221 | 0.008 | 0.355 | 0.000 | 0.288 | 0.004 | -0.049 | -0.314 | 0.266 |
| Toluene | 0.146 | 0.018 | 0.130 | 0.021 | 0.138 | 0.019 | -0.012 | -0.249 | 0.237 |
| 2,4,5-trichlorophenoxy acetic acid | 0.150 | 0.003 | 0.129 | 0.002 | 0.121 | 0.003 | -0.046 | -0.247 | 0.200 |
| Alachlor | 0.125 | -0.003 | 0.783 | -0.001 | 0.402 | 0.000 | -0.025 | -0.252 | 0.227 |
| Atrazine | 0.134 | 0.008 | 0.822 | 0.000 | 0.465 | 0.005 | -0.028 | -0.247 | 0.219 |
| Carbofuran | 0.118 | 0.005 | 0.137 | 0.006 | 0.102 | 0.006 | -0.016 | -0.224 | 0.208 |
| Dicamba | 0.170 | 0.004 | 0.118 | 0.006 | 0.130 | 0.019 | -0.052 | -0.260 | 0.209 |
| Glyphosate | 0.275 | 0.019 | 0.290 | 0.001 | 0.159 | 0.010 | -0.005 | -0.251 | 0.246 |
| Metolachlor | 0.131 | -0.003 | 0.776 | -0.004 | 0.401 | 0.000 | -0.027 | -0.253 | 0.226 |
| Simazine | 0.135 | 0.009 | 0.846 | 0.000 | 0.477 | 0.005 | -0.027 | -0.247 | 0.220 |
| Bromoform | 0.317 | 0.024 | 0.843 | -0.001 | 0.578 | 0.048 | -0.085 | -0.290 | 0.205 |
| 1,1,1-Trichloroethane | 0.352 | 0.021 | 0.298 | 0.014 | 0.325 | 0.018 | -0.053 | -0.317 | 0.265 |
| Bromodichloromethane | 0.471 | 0.039 | 0.840 | 0.000 | 0.655 | 0.052 | -0.069 | -0.305 | 0.236 |
| 1,1-Dichloroethane | 0.265 | 0.058 | 0.289 | 0.116 | 0.257 | 0.087 | -0.025 | -0.274 | 0.249 |
| Isophorone | 0.340 | 0.009 | 0.221 | 0.006 | 0.281 | 0.009 | -0.055 | -0.250 | 0.195 |
| 1,2-Dichloropropane | 0.326 | 0.022 | 0.786 | -0.004 | 0.550 | 0.012 | -0.009 | -0.308 | 0.300 |
| 1,1,2-Trichloroethane | 0.299 | 0.029 | 0.384 | 0.016 | 0.341 | 0.023 | -0.037 | -0.317 | 0.280 |
| Trichloroethylene | 0.260 | 0.044 | 0.728 | 0.020 | 0.477 | 0.032 | -0.036 | -0.268 | 0.232 |
| Diquat | 0.087 | 0.014 | 0.066 | 0.019 | 0.076 | 0.016 | -0.145 | -0.315 | 0.170 |
| Dinoseb | 0.121 | 0.006 | 0.223 | 0.002 | 0.141 | 0.004 | -0.106 | -0.269 | 0.164 |
| o-Chlorotoluene | 0.226 | 0.023 | 0.148 | 0.017 | 0.165 | 0.021 | -0.022 | -0.254 | 0.231 |
| o-Dichlorobenzene | 0.196 | 0.031 | 0.128 | 0.035 | 0.155 | 0.033 | -0.034 | -0.262 | 0.228 |
| Dibromochloropropane | 0.425 | 0.010 | 0.540 | -0.007 | 0.483 | 0.007 | -0.052 | -0.294 | 0.242 |
| 1,2,3-Trichloropropane | 0.265 | 0.026 | 0.551 | -0.007 | 0.398 | 0.014 | -0.027 | -0.313 | 0.286 |
| Ethyl benzene | 0.146 | 0.009 | 0.148 | 0.010 | 0.145 | 0.009 | -0.012 | -0.249 | 0.238 |
| Styrene | 0.169 | 0.030 | 0.170 | 0.027 | 0.169 | 0.029 | -0.046 | -0.235 | 0.190 |
| p-Xylene | 0.129 | 0.030 | 0.122 | 0.020 | 0.099 | 0.025 | -0.011 | -0.239 | 0.228 |
| p-Chlorotoluene | 0.225 | 0.029 | 0.124 | 0.020 | 0.160 | 0.024 | -0.023 | -0.249 | 0.225 |
| 1,2-Dibromoethane | 0.403 | 0.026 | 0.480 | 0.006 | 0.442 | 0.016 | -0.044 | -0.291 | 0.247 |
| 1,3,5-Trichlorobenzene | 0.218 | 0.031 | 0.120 | 0.028 | 0.166 | 0.030 | -0.045 | -0.271 | 0.226 |
| Bromobenzene | 0.319 | 0.034 | 0.907 | 0.000 | 0.613 | 0.017 | -0.025 | -0.255 | 0.230 |
| Chlorobenzene | 0.256 | 0.037 | 0.131 | 0.038 | 0.177 | 0.043 | -0.025 | -0.258 | 0.234 |
| 2,4-Dinitrotoluene | 0.137 | 0.016 | 0.220 | 0.009 | 0.139 | 0.013 | -0.112 | -0.293 | 0.181 |
| 1,3-Dichloropropane | 0.337 | 0.020 | 0.764 | 0.003 | 0.550 | 0.015 | 0.006 | -0.307 | 0.314 |
| 1,1-Dichloropropene | 0.246 | 0.030 | 0.889 | -0.035 | 0.560 | 0.025 | -0.013 | -0.261 | 0.247 |
| 1,1,1,2-Tetrachloroethane | 0.352 | 0.017 | 0.844 | -0.024 | 0.490 | 0.021 | -0.056 | -0.321 | 0.265 |
| tert-Butyl methyl ether | 0.275 | 0.025 | 0.134 | 0.009 | 0.142 | 0.024 | 0.040 | -0.257 | 0.297 |
| Picloram | 0.158 | 0.011 | 0.125 | 0.026 | 0.106 | 0.028 | -0.077 | -0.253 | 0.175 |
| Metribuzin | 0.167 | 0.008 | 0.142 | 0.006 | 0.146 | 0.007 | -0.070 | -0.247 | 0.178 |
| Cyanazine | 0.172 | -0.001 | 0.840 | 0.000 | 0.468 | 0.000 | -0.035 | -0.254 | 0.218 |
| Dibromochloromethane | 0.387 | 0.038 | 0.401 | 0.042 | 0.394 | 0.040 | -0.078 | -0.296 | 0.218 |
| trans-1,2-Dichloroethylene | 0.297 | 0.050 | 0.797 | 0.001 | 0.547 | 0.025 | -0.026 | -0.267 | 0.241 |
| cis-1,2-Dichloroethylene | 0.288 | 0.052 | 0.838 | 0.005 | 0.563 | 0.028 | -0.022 | -0.269 | 0.247 |
| Aldicarb | 0.390 | 0.005 | 0.779 | -0.018 | 0.585 | 0.002 | -0.032 | -0.240 | 0.208 |
| Oxamyl | 0.274 | 0.009 | 0.153 | 0.006 | 0.213 | 0.008 | -0.048 | -0.248 | 0.201 |

**S2 Table. Information on Freundlich parameters and 31 molecular parameters for 47 organic compounds (part 3)**

| **Name** | **BO(C-C)_max_** | **BO(C-C)_min_** | **BO(C-H)_max_** | **BO(C-H)_min_** | **Wiberg(C-C)_max_** | **Wiberg(C-C)_min_** | **Wiberg(C-H)_max_** | **Wiberg(C-H)_min_** |
| --- | --- | --- | --- | --- | --- | --- | --- | --- |
| 1,2-Dichloroethane | -0.544 | -0.693 | -0.544 | -0.547 | 3.893 | 1.083 | 0.951 | 0.947 |
| Benzene | -0.517 | -0.956 | -0.517 | -0.517 | 3.963 | 3.963 | 0.955 | 0.955 |
| Lindane | -0.557 | -0.703 | -0.557 | -0.560 | 3.973 | 1.126 | 0.938 | 0.932 |
| Toluene | -0.495 | -0.950 | -0.495 | -0.515 | 4.005 | 3.883 | 0.957 | 0.954 |
| 2,4,5-trichlorophenoxy acetic acid | -0.532 | -1.530 | -0.532 | -0.551 | 4.017 | 1.219 | 0.964 | 0.939 |
| Alachlor | -0.486 | -1.476 | -0.486 | -0.550 | 4.005 | 1.104 | 0.974 | 0.941 |
| Atrazine | -0.496 | -1.239 | -0.496 | -0.663 | 4.007 | 1.222 | 0.962 | 0.835 |
| Carbofuran | -0.500 | -1.472 | -0.500 | -0.671 | 3.997 | 3.818 | 0.962 | 0.826 |
| Dicamba | -0.530 | -1.542 | -0.530 | -0.773 | 4.015 | 1.224 | 0.969 | 0.746 |
| Glyphosate | -0.525 | -1.525 | -0.525 | -0.797 | 3.895 | 3.824 | 0.958 | 0.729 |
| Metolachlor | -0.488 | -1.459 | -0.488 | -0.554 | 4.015 | 1.103 | 0.976 | 0.941 |
| Simazine | -0.503 | -1.239 | -0.503 | -0.663 | 4.010 | 1.223 | 0.963 | 0.835 |
| Bromoform | -0.602 | -0.669 | -0.602 | -0.602 | 3.919 | 3.919 | 0.949 | 0.949 |
| 1,1,1-Trichloroethane | -0.526 | -0.733 | -0.526 | -0.526 | 4.071 | 1.220 | 0.945 | 0.945 |
| Bromodichloromethane | -0.604 | -0.741 | -0.604 | -0.604 | 3.949 | 1.224 | 0.949 | 0.949 |
| 1,1-Dichloroethane | -0.544 | -1.158 | -0.544 | -0.544 | 4.020 | 1.265 | 0.949 | 0.949 |
| Isophorone | -0.490 | -1.442 | -0.490 | -0.515 | 4.017 | 3.880 | 0.960 | 0.946 |
| 1,2-Dichloropropane | -0.509 | -0.685 | -0.509 | -0.544 | 3.957 | 1.085 | 0.954 | 0.948 |
| 1,1,2-Trichloroethane | -0.554 | -0.722 | -0.554 | -0.576 | 3.967 | 1.103 | 0.943 | 0.943 |
| Trichloroethylene | -0.572 | -1.199 | -0.572 | -0.572 | 4.024 | 1.242 | 0.940 | 0.940 |
| Diquat | -0.568 | -1.343 | -0.568 | -0.598 | 3.950 | 3.807 | 0.938 | 0.932 |
| Dinoseb | -0.489 | -1.610 | -0.489 | -0.561 | 4.009 | 3.882 | 0.969 | 0.753 |
| o-Chlorotoluene | -0.502 | -1.005 | -0.502 | -0.528 | 4.006 | 1.196 | 0.955 | 0.949 |
| o-Dichlorobenzene | -0.529 | -1.071 | -0.529 | -0.535 | 4.014 | 1.219 | 0.950 | 0.946 |
| Dibromochloropropane | -0.547 | -0.704 | -0.547 | -0.552 | 3.968 | 1.094 | 0.946 | 0.939 |
| 1,2,3-Trichloropropane | -0.546 | -0.696 | -0.546 | -0.551 | 3.962 | 1.093 | 0.948 | 0.942 |
| Ethyl benzene | -0.486 | -0.949 | -0.486 | -0.515 | 4.010 | 3.890 | 0.963 | 0.956 |
| Styrene | -0.510 | -1.031 | -0.510 | -0.526 | 4.005 | 3.933 | 0.966 | 0.955 |
| p-Xylene | -0.494 | -0.943 | -0.494 | -0.510 | 4.004 | 3.883 | 0.958 | 0.955 |
| p-Chlorotoluene | -0.497 | -1.014 | -0.497 | -0.529 | 4.006 | 1.188 | 0.955 | 0.949 |
| 1,2-Dibromoethane | -0.549 | -0.702 | -0.549 | -0.549 | 3.898 | 3.898 | 0.950 | 0.950 |
| 1,3,5-Trichlorobenzene | -0.547 | -1.049 | -0.547 | -0.547 | 4.016 | 1.220 | 0.939 | 0.939 |
| Bromobenzene | -0.523 | -1.022 | -0.523 | -0.530 | 4.017 | 3.955 | 0.953 | 0.948 |
| Chlorobenzene | -0.523 | -1.020 | -0.523 | -0.531 | 4.010 | 1.194 | 0.953 | 0.948 |
| 2,4-Dinitrotoluene | -0.511 | -1.606 | -0.511 | -0.559 | 3.993 | 3.865 | 0.955 | 0.926 |
| 1,3-Dichloropropane | -0.508 | -0.658 | -0.508 | -0.538 | 3.928 | 1.072 | 0.954 | 0.948 |
| 1,1-Dichloropropene | -0.506 | -1.138 | -0.506 | -0.526 | 4.004 | 1.240 | 0.953 | 0.948 |
| 1,1,1,2-Tetrachloroethane | -0.562 | -0.751 | -0.562 | -0.562 | 4.083 | 1.124 | 0.939 | 0.939 |
| tert-Butyl methyl ether | -0.492 | -0.799 | -0.492 | -0.515 | 3.916 | 3.792 | 0.976 | 0.956 |
| Picloram | -0.691 | -1.533 | -0.691 | -0.772 | 4.016 | 1.239 | 0.825 | 0.747 |
| Metribuzin | -0.491 | -1.486 | -0.491 | -0.664 | 4.021 | 3.845 | 0.961 | 0.854 |
| Cyanazine | -0.503 | -1.250 | -0.503 | -0.671 | 4.006 | 1.233 | 0.961 | 0.820 |
| Dibromochloromethane | -0.603 | -0.747 | -0.603 | -0.603 | 3.934 | 1.236 | 0.949 | 0.949 |
| trans-1,2-Dichloroethylene | -0.571 | -1.159 | -0.571 | -0.571 | 3.946 | 1.215 | 0.947 | 0.947 |
| cis-1,2-Dichloroethylene | -0.565 | -1.155 | -0.565 | -0.565 | 3.950 | 1.230 | 0.944 | 0.944 |
| Aldicarb | -0.500 | -1.476 | -0.500 | -0.671 | 4.036 | 3.818 | 0.963 | 0.827 |
| Oxamyl | -0.522 | -1.485 | -0.522 | -0.674 | 4.009 | 3.805 | 0.963 | 0.826 |

**S2 Table. Information on Freundlich parameters and 31 molecular parameters for 47 organic compounds (part 4)**

| **Name** | **∑q(O)** | **∑q(N)** | **∑q(O+N)** | **∑q(C)** | **∑q(H)** | **∑q(H)/N_H_** | **∑q(-)/N_C_** | **MW** |
| --- | --- | --- | --- | --- | --- | --- | --- | --- |
| 1,2-Dichloroethane | 0.000 | 0.000 | 0.000 | -0.242 | 0.242 | 0.061 | -0.061 | 98.954 |
| Benzene | 0.000 | 0.000 | 0.000 | -0.271 | 0.271 | 0.045 | -0.045 | 78.114 |
| Lindane | 0.000 | 0.000 | 0.000 | -0.315 | 0.315 | 0.053 | -0.026 | 290.814 |
| Toluene | 0.000 | 0.000 | 0.000 | -0.329 | 0.329 | 0.041 | -0.047 | 92.141 |
| 2,4,5-trichlorophenoxy acetic acid | -0.537 | 0.000 | -0.537 | 0.132 | 0.405 | 0.058 | 0.011 | 269.502 |
| Alachlor | -0.499 | -0.028 | -0.527 | -0.236 | 0.763 | 0.038 | -0.016 | 269.769 |
| Atrazine | 0.000 | -0.817 | -0.817 | 0.121 | 0.696 | 0.050 | 0.013 | 215.685 |
| Carbofuran | -0.642 | -0.078 | -0.720 | -0.019 | 0.739 | 0.049 | -0.002 | 221.256 |
| Dicamba | -0.606 | 0.000 | -0.606 | 0.157 | 0.448 | 0.075 | 0.016 | 221.033 |
| Glyphosate | -1.403 | -0.152 | -1.555 | 0.176 | 0.898 | 0.112 | 0.059 | 169.073 |
| Metolachlor | -0.511 | -0.024 | -0.535 | -0.294 | 0.830 | 0.038 | -0.018 | 283.796 |
| Simazine | 0.000 | -0.825 | -0.825 | 0.180 | 0.645 | 0.054 | 0.023 | 201.658 |
| Bromoform | 0.000 | 0.000 | 0.000 | 0.034 | 0.069 | 0.069 | 0.034 | 252.731 |
| 1,1,1-Trichloroethane | 0.000 | 0.000 | 0.000 | -0.155 | 0.155 | 0.052 | -0.031 | 133.396 |
| Bromodichloromethane | 0.000 | 0.000 | 0.000 | -0.032 | 0.072 | 0.072 | -0.011 | 163.823 |
| 1,1-Dichloroethane | 0.000 | 0.000 | 0.000 | -0.127 | 0.127 | 0.064 | -0.032 | 96.938 |
| Isophorone | -0.341 | 0.000 | -0.341 | -0.197 | 0.538 | 0.038 | -0.022 | 138.210 |
| 1,2-Dichloropropane | 0.000 | 0.000 | 0.000 | -0.297 | 0.297 | 0.050 | -0.059 | 112.981 |
| 1,1,2-Trichloroethane | 0.000 | 0.000 | 0.000 | -0.205 | 0.205 | 0.068 | -0.041 | 133.396 |
| Trichloroethylene | 0.000 | 0.000 | 0.000 | -0.072 | 0.072 | 0.072 | -0.015 | 131.380 |
| Diquat | 0.000 | 0.154 | 0.154 | 0.725 | 1.121 | 0.093 | 0.060 | 184.242 |
| Dinoseb | -1.062 | 0.502 | -0.560 | -0.059 | 0.619 | 0.052 | -0.006 | 240.215 |
| o-Chlorotoluene | 0.000 | 0.000 | 0.000 | -0.313 | 0.313 | 0.045 | -0.039 | 126.583 |
| o-Dichlorobenzene | 0.000 | 0.000 | 0.000 | -0.228 | 0.228 | 0.057 | -0.029 | 146.998 |
| Dibromochloropropane | 0.000 | 0.000 | 0.000 | -0.112 | 0.269 | 0.054 | -0.028 | 236.331 |
| 1,2,3-Trichloropropane | 0.000 | 0.000 | 0.000 | -0.274 | 0.274 | 0.055 | -0.046 | 147.423 |
| Ethyl benzene | 0.000 | 0.000 | 0.000 | -0.370 | 0.370 | 0.037 | -0.046 | 106.168 |
| Styrene | 0.000 | 0.000 | 0.000 | -0.353 | 0.353 | 0.044 | -0.044 | 104.152 |
| p-Xylene | 0.000 | 0.000 | 0.000 | -0.385 | 0.385 | 0.039 | -0.048 | 106.168 |
| p-Chlorotoluene | 0.000 | 0.000 | 0.000 | -0.323 | 0.323 | 0.046 | -0.040 | 126.583 |
| 1,2-Dibromoethane | 0.000 | 0.000 | 0.000 | -0.022 | 0.215 | 0.054 | -0.011 | 187.862 |
| 1,3,5-Trichlorobenzene | 0.000 | 0.000 | 0.000 | -0.180 | 0.180 | 0.060 | -0.020 | 181.440 |
| Bromobenzene | 0.000 | 0.000 | 0.000 | -0.193 | 0.259 | 0.052 | -0.032 | 157.010 |
| Chlorobenzene | 0.000 | 0.000 | 0.000 | -0.260 | 0.260 | 0.052 | -0.037 | 112.556 |
| 2,4-Dinitrotoluene | -0.864 | 0.509 | -0.355 | 0.024 | 0.332 | 0.055 | 0.003 | 182.135 |
| 1,3-Dichloropropane | 0.000 | 0.000 | 0.000 | -0.294 | 0.294 | 0.049 | -0.059 | 112.981 |
| 1,1-Dichloropropene | 0.000 | 0.000 | 0.000 | -0.189 | 0.189 | 0.047 | -0.038 | 110.965 |
| 1,1,1,2-Tetrachloroethane | 0.000 | 0.000 | 0.000 | -0.124 | 0.124 | 0.062 | -0.021 | 167.838 |
| tert-Butyl methyl ether | -0.193 | 0.000 | -0.193 | -0.199 | 0.392 | 0.033 | -0.040 | 88.150 |
| Picloram | -0.461 | -0.292 | -0.753 | 0.296 | 0.456 | 0.152 | 0.033 | 241.452 |
| Metribuzin | -0.291 | -0.357 | -0.648 | -0.020 | 0.646 | 0.046 | -0.003 | 214.287 |
| Cyanazine | 0.000 | -1.052 | -1.052 | 0.307 | 0.745 | 0.057 | 0.031 | 240.695 |
| Dibromochloromethane | 0.000 | 0.000 | 0.000 | 0.004 | 0.070 | 0.070 | 0.002 | 208.277 |
| trans-1,2-Dichloroethylene | 0.000 | 0.000 | 0.000 | -0.136 | 0.136 | 0.068 | -0.034 | 96.938 |
| cis-1,2-Dichloroethylene | 0.000 | 0.000 | 0.000 | -0.145 | 0.145 | 0.072 | -0.036 | 96.938 |
| Aldicarb | -0.471 | -0.158 | -0.630 | -0.007 | 0.682 | 0.049 | -0.001 | 190.261 |
| Oxamyl | -0.793 | -0.189 | -0.982 | 0.247 | 0.712 | 0.055 | 0.035 | 219.259 |
