# Supplementary material for: Quantitative structure activity relationship (QSAR) modeling for adsorption of organic compounds by activated carbon based on Freundlich adsorption isotherm
Source: PLoS One. 2025 Dec 15;20(12):e0338483. doi: 10.1371/journal.pone.0338483 (PMC12704836; doi:10.1371/journal.pone.0338483)
Supplement: S3 Table — (DOCX) [file pone.0338483.s003.docx]

***Supporting information***

**S3 Table. Meaning of evaluation indexes**

| **Name** | **Name** | **Connotation** |
| --- | --- | --- |
| 1 | R^2^ | regression coefficient |
| 2 | R_adj_^2^ | adjusted regression coefficient |
| 3 | R^2^-R_adj_^2^ | difference between regression coefficient and adjusted regression coefficient |
| 4 | R_ext_^2^ | external validation coefficient |
| 5 | SD | standard deviation |
| 6 | RMSE | root mean square error |
| 7 | t | t test |
| 8 | F | Fisher test |
| 9 | Sig. | significance |
| 10 | VIF | variance inflation factor test |
| 11 | q^2^ | internal validation coefficient |
| 12 | Q_ext_^2^ | external validation coefficient |
| 13 | Q_LOO_^2^ | Leave-one-out cross-validation coefficient |
| 14 | Q_LMO_^2^ | Leave-one-out cross-validation coefficient |
| 15 | R^2^_yrand_ | Y regression coefficient after randomized upset |
| 16 | Q^2^_yrand_ | Y external validation after randomized upset |
| 17 | LOF | the density deviation between a computed data point and its neighbors |
| 18 | K_xx_ | Overall correlation between descriptors |
| 19 | Delta K | the overall correlation between descriptors; Delta K is the difference between K_xx_ and K_xy_ |
| 20 | RMSE_tr_ | the root of the mean squared error computed for the training set |
| 21 | MAE_tr_ | the mean in calibration computed for the training set absolute error |
| 22 | RSS_tr_ | the sum of squares of residuals in the correction for the training set |
| 23 | CCC_tr_ | the consistency correlation coefficient of the training set |
